# Radioligand Therapy in Cancer Management: A Global Perspective

**DOI:** 10.3390/cancers17213412

**Published:** 2025-10-23

**Authors:** Gaia Ninatti, Sze Ting Lee, Andrew M. Scott

**Affiliations:** 1 Olivia Newton-John Cancer Research Institute, Heidelberg, Melbourne, VIC 3084, Australia; 2 Department of Molecular Imaging and Therapy, Austin Health, Heidelberg, Melbourne, VIC 3084, Australia; 3 School of Medicine and Surgery, University of Milano-Bicocca, 20900 Monza, Italy; 4 School of Cancer Medicine, La Trobe University, Bundoora, Melbourne, VIC 3086, Australia; 5 School of Health and Biomedicine, RMIT University, Melbourne, VIC 3000, Australia; 6 Faculty of Medicine, University of Melbourne, Parkville, Melbourne, VIC 3052, Australia

**Keywords:** radioligand therapy, theranostics, [^177^Lu]Lu-DOTA-TATE, [^177^Lu]Lu-PSMA-617, neuroendocrine tumours, prostate cancer

## Abstract

**Simple Summary:**

Radioligand therapy is an emerging treatment strategy in oncology that delivers targeted radiation to cancer cells while sparing surrounding healthy tissues. Two radiopharmaceuticals are already approved for patients with advanced neuroendocrine tumours and prostate cancer, and many more are being tested in clinical trials for different cancers. In this review, we outline the mechanisms of action of radioligand therapy, summarise current clinical evidence, and discuss promising new targets under investigation. Moreover, we describe major challenges that limit access to this treatment in many countries, including shortages in production, limited specialised workforce, and fragmented regulatory pathways. The aim of this review is to provide a comprehensive overview of the field and to highlight the main steps required to advance radioligand therapy in oncology.

**Abstract:**

Radioligand therapy (RLT) is a targeted treatment modality that combines a tumour-specific ligand with a therapeutic radionuclide. Once administered, the radiopharmaceutical binds selectively to cancer-associated targets, delivering cytotoxic radiation directly to tumour cells while sparing surrounding tissues. Two RLT agents, [^177^Lu]Lu-DOTA-TATE (Lutathera^®^) and [^177^Lu]Lu-PSMA-617 (Pluvicto^®^), have received regulatory approval for the treatment of advanced gastroenteropancreatic neuroendocrine tumours and metastatic castration-resistant prostate cancer, respectively. As of July 2025, more than 400 clinical trials are registered, exploring novel molecular targets such as FAP, CAIX, and GRPR, as well as alternative radionuclides and combination regimens in both solid and haematologic malignancies. In this review, we describe the design principles and mechanisms of action of RLT, summarise clinical evidence for approved and emerging radiopharmaceuticals, and discuss current global disparities in access and availability. Finally, we outline the main clinical challenges, including fixed dosing regimens, resistance, toxicity, and variability in patient selection and response assessment. Continued research to optimise radiopharmaceutical design, together with investment in infrastructure, workforce capacity, and international collaboration, will be essential to expand access and realise the full potential of RLT as a leading treatment strategy in modern oncology.

## 1. Introduction

Cancer therapy has advanced considerably over the past two decades. Nevertheless, a significant proportion of patients with solid and haematologic malignancies continue to face limited therapeutic options, particularly in advanced stages of disease. Chemotherapy remains a mainstay of cancer treatment, but its lack of tumour specificity and associated systemic toxicities have long driven the search for more targeted and better tolerated approaches.

The introduction of immunotherapy and tyrosine kinase inhibitors in the early 2000s transformed the management of several tumour types [1,2]. By targeting immune checkpoints or dysregulated signalling pathways, these agents have demonstrated superior efficacy and safety compared to traditional cytotoxic therapies. However, resistance, suboptimal responses, and dose-limiting toxicities are common, and most patients eventually progress.

To address these limitations, a range of targeted strategies has entered clinical development, including antibody-drug conjugates, bispecific and multispecific antibodies, CAR-T cells, and radioligand therapy (RLT) [3]. Among these modalities, RLT has emerged as a particularly promising approach, combining molecular precision with the cytotoxic effect of ionising radiation [4]. RLT is based on the administration of radiopharmaceuticals composed of a targeting ligand linked to a therapeutic radionuclide. Following administration, the ligand binds selectively to tumour-associated targets, delivering radiation to cancer cells while sparing surrounding normal tissue.

Although the field is still evolving, RLT has already entered clinical practice. Two agents, [^177^Lu]Lu-DOTA-TATE and [^177^Lu]Lu-PSMA-617, are approved for patients with advanced neuroendocrine tumours and prostate cancer, respectively, with favourable efficacy and safety demonstrated in randomised clinical trials. Building on these results, multiple investigational agents are now under evaluation, exploring novel molecular targets, alternative radionuclides, and combination strategies in multiple disease settings.

The present review summarises the current state of RLT, including design principles, mechanism of action, clinical applications, and global availability, and discusses the challenges that must be addressed for wider implementation and clinical optimisation.

## 2. Radioligand Therapy: Design and Mechanism of Action

### 2.1. Design

RLT agents consist of three main components: a ligand, a linker, and a radionuclide. The ligand binds selectively to tumour-associated targets, including receptors, surface antigens, transporters, or enzymes expressed on cancer cells or within the tumour microenvironment. The linker connects the ligand to the radionuclide and maintains the structural integrity and stability of the radiopharmaceutical. The radionuclide is a radioactive isotope that emits high-energy radiation capable of inducing irreparable DNA damage, leading to tumour cell death. Each component contributes to the efficacy, tumour selectivity, and safety of the radiopharmaceutical, and their optimisation is central to RLT development.

**Figure 1:**
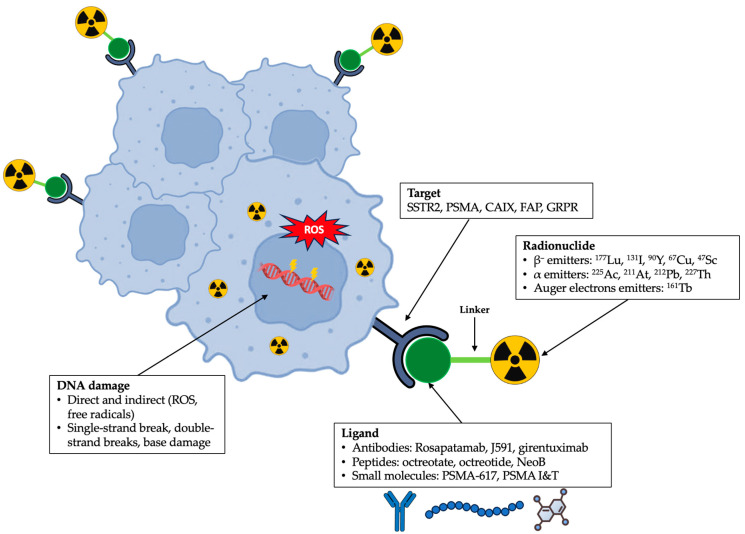
Schematic representation of radioligand therapy agents, illustrating their components and mechanism of action.

Figure 1 provides an overview of the components of RLT agents and their mechanism of action.

#### 2.1.1. Ligand

The ligand is the component of a RLT agent that recognises and binds to a tumour-associated target. Ligands used in RLT are broadly classified into three main categories: antibodies, peptides, or small molecules. Each has distinct pharmacokinetic and pharmacodynamic properties that influence binding affinity, tumour penetration, clearance, and ultimately efficacy and toxicity [5].

Antibodies have high specificity and long circulation times, resulting in improved tumour retention and radiation delivery. However, their high molecular weight limits tumour penetration and slows clearance from non-target tissues [6]. Small molecules are easier to synthesise and radiolabel, penetrate tumours efficiently due to their low molecular weight, and clear rapidly from the circulation; however, their shorter circulation time and shorter tumour retention may reduce overall radiation delivery compared with antibodies [7]. Peptides have intermediate characteristics, combining effective tumour targeting with faster clearance than antibodies, and can be chemically modified to improve stability and affinity [8,9]. The choice of the ligand should be tailored to tumour biology, the expression pattern of the target antigen, and patient-specific factors such as tumour burden and baseline organ function. Finding the right balance between on-target efficacy and off-target safety is central to optimising treatment outcomes.

Target selection is equally important. An ideal target for RLT should be abundantly and homogeneously expressed on tumour cells or within the tumour microenvironment, with consistent expression not only within individual lesions, but also between metastatic sites. At the same time, expression in normal tissues should be minimal to reduce the risk of off-target toxicity.

#### 2.1.2. Linker

The linker connects the targeting ligand to the radionuclide and is an essential element of RLT agents [10]. It contributes to the chemical stability of the radiopharmaceutical and influences tumour uptake, biodistribution, and systemic clearance, directly affecting efficacy and safety.

Linkers are generally classified into three categories: cleavable, non-cleavable, and pharmacokinetic-modifying. Cleavable linkers are designed to release the radionuclide in response to specific triggers, such as enzymatic activity, allowing selective delivery and limiting accumulation in non-target tissues, particularly the kidneys [11,12]. Non-cleavable linkers remain stable in circulation and preserve the integrity of the radiopharmaceutical until internalisation by target cells. Pharmacokinetic-modifying linkers are designed to optimise circulation time, biodistribution, and excretion, often improving clearance and reducing off-target exposure.

The selection of an appropriate linker should take into account tumour biology, the properties of the targeting ligand, the physical characteristics of the radionuclide, and patient-specific factors.

#### 2.1.3. Radionuclide

The radionuclide is the therapeutic component of a RLT agent, exerting its cytotoxic effect through the emission of ionising radiation. Radionuclides used in RLT emit β-particles, α-particles, Auger electrons, or a combination of these, each with different physical properties that determine their biological behaviour [13].

To date, β-emitters are the most commonly used in clinical practice. β-particles have relatively low linear energy transfer (LET) and a path length in tissues of up to several millimetres, allowing irradiation of both the targeted tumour cell and adjacent cells (crossfire effect). α-particles have high LET and a short tissue range of up to 100 micrometres, causing complex DNA double-strand breaks. Auger electrons have intermediate LET compared with α- and β-particles, and an extremely short range, typically less than one micrometre. For this reason, Auger electrons emitting radiopharmaceuticals need to be internalised and localise in close proximity to the nucleus to exert their cytotoxic effect.

Radionuclide selection should be based on tumour features, including lesion size, heterogeneity, and radiosensitivity, as well as patient characteristics. α-particles and Auger electrons, with their high LET and short range, may be more suitable for micrometastatic or radioresistant disease, while β-particles may be preferred in larger or more heterogeneous lesions.

### 2.2. Mechanism of Action

RLT agents are generally administered intravenously and circulate in the bloodstream until they reach their target, typically a receptor or antigen expressed on tumour cells or within the tumour microenvironment. Following binding, internalisation may occur depending on the nature of the ligand and the target. Small molecules and peptides are usually internalised through receptor-mediated endocytosis [14,15], resulting in intracellular delivery of the radionuclide. In contrast, radiolabelled antibodies may remain bound to surface antigens for prolonged periods without internalisation but can still exert their cytotoxic effects through emission of radiation in close proximity to the cell membrane [16,17]. Once the radiopharmaceutical is internalised or bound to a surface antigen, the radionuclide emits ionising radiation that induces cellular damage through multiple mechanisms, including single- and double-strand DNA breaks, generation of reactive oxygen species, and damage to cellular organelles and membranes. The extent and nature of cellular damage depend on the energy and range of the emitted particles, as well as their distance from the nucleus, and may result in cell death, most commonly by apoptosis or necrosis. The cytotoxic effect may be enhanced by the bystander effect, in which non-irradiated neighbouring cells are damaged in response to molecular signals or mediators such as cytokines, chemokines, and reactive oxygen species released from irradiated cells.

## 3. Clinical Applications of Radioligand Therapy

The most extensively studied and widely used RLT agents in clinical practice target somatostatin receptors (SSTRs) and prostate-specific membrane antigen (PSMA). Two radiopharmaceuticals in these categories have received regulatory approval from both the European Medicines Agency (EMA) and the U.S. Food and Drug Administration (FDA): [^177^Lu]Lu-DOTA-TATE (Lutathera^®^), which targets SSTRs, and [^177^Lu]Lu-PSMA-617 (Pluvicto^®^), which targets PSMA. Table 1 provides an overview of their design, approved indications, dosing regimens, and adverse reactions.

**Figure 2:**
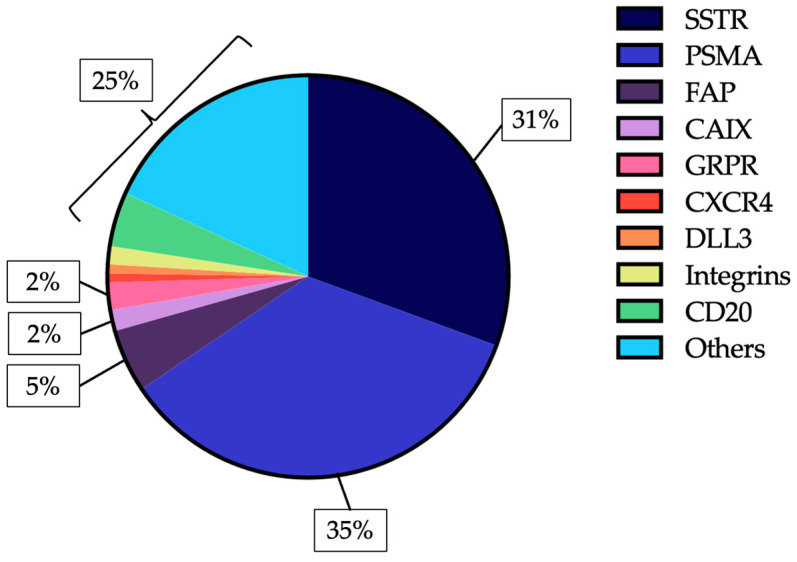
Relative frequency of molecular targets among 411 RLT trials registered on ClinicalTrials.gov as of 5 July 2025.

As of 5 July 2025, more than 400 clinical trials investigating RLT were registered on ClinicalTrials.gov. SSTR- and PSMA-targeted agents account for approximately 65% of these studies. Among the other targets most frequently investigated in solid tumours are fibroblast activation protein (FAP), carbonic anhydrase IX (CAIX), and gastrin-releasing peptide receptor (GRPR). Although no RLT agent directed against these targets has yet received regulatory approval, multiple early-phase trials have been completed or are ongoing, with preliminary findings suggesting promising therapeutic activity in several tumour types. Additional investigational targets include chemokine receptor 4 (CXCR4), delta-like ligand 3 (DLL3), folate receptor α (FRα), six-transmembrane epithelial antigen of the prostate 2 (STEAP2), insulin-like growth factor 1 receptor (IGF-1R), melanocortin 1 receptor (MC1R), nectin-4, (human epidermal growth factor receptor 2) HER2, and various cluster of differentiation (CD) antigens. Details of the search strategy and inclusion criteria are provided in the Supplementary Materials. Figure 2 summarises the most commonly explored targets in registered RLT trials.

### 3.1. SSTR-Targeted Radioligand Therapy

SSTR-targeted radiopharmaceuticals bind selectively to somatostatin receptors, which are overexpressed in several malignancies, most notably in neuroendocrine tumours (NETs), but also in paragangliomas, pheochromocytomas, meningiomas, gliomas, and some breast cancer subtypes.

The first SSTR-targeted RLT agent to receive regulatory approval was [^177^Lu]Lu-DOTATATE (Lutathera^®^), a radiolabelled peptide that delivers β-emitting lutetium-177 to tumour cells expressing somatostatin receptors, with particular affinity for subtype 2 (SSTR2) and, to a lesser extent, subtype 5 (SSTR5) [18].

[^177^Lu]Lu-DOTA-TATE (Lutathera^®^) was approved by the European Medicines Agency (EMA) in September 2017 and the U.S. Food and Drug Administration (FDA) in January 2018 for the treatment of progressive, SSTR-positive, unresectable or metastatic, well-differentiated (G1-G2) gastroenteropancreatic NETs (GEP NETs). It has since received approval in several additional countries and is increasingly used in routine clinical practice worldwide, including incorporation into treatment guidelines.

Regulatory approval was based on results from the phase 3 NETTER-1 trial and earlier supporting studies. NETTER-1 enrolled patients with progressive, unresectable or metastatic G1-G2 midgut NETs and demonstrated significantly longer progression-free survival (PFS) (28.4 vs. 8.4 months, *p* < 0.001) and higher objective response rates (ORRs) (18% vs. 3%, *p* < 0.001) with [^177^Lu]Lu-DOTA-TATE combined with long-acting octreotide compared with high-dose long-acting octreotide alone [19]. The final overall survival (OS) analysis showed a median OS benefit of 11.7 months in the [^177^Lu]Lu-DOTA-TATE arm; however, the difference did not reach statistical significance (*p* = 0.30) [20]. Although interpretation is limited by the high rate of crossover from the control to the [^177^Lu]Lu-DOTA-TATE arm, the magnitude of the difference supports a clinically meaningful survival benefit consistent with the PFS and ORR data.

In April 2024, the FDA expanded the indication for [^177^Lu]Lu-DOTA-TATE (Lutathera^®^) to include paediatric patients aged 12 years and older with advanced SSTR-positive, G1-G2 GEP NETs, based on the phase 2 NETTER-P trial, which confirmed safety and preliminary efficacy in this population [21].

Evidence for efficacy in pancreatic NETs was initially provided by the NETTER-R and OCCLURANDOM trials. NETTER-R, a retrospective study published in 2022, reported a median PFS of 24.5 months, an ORR of 40.3%, and median OS of 41.4 months with [^177^Lu]Lu-DOTA-TATE [22]. OCCLURANDOM, a phase 2 randomised trial in pretreated, progressive, advanced SSTR-positive G1-G2 pancreatic NETs, demonstrated longer PFS (20.7 vs. 11.0 months) and higher ORRs (63% vs. 30%) with [^177^Lu]Lu-DOTA-TATE compared with sunitinib [23].

An ongoing phase 3 trial has recently consolidated evidence for the value of SSTR-targeted RLT in progressive advanced GEP NETs. Interim results from the COMPETE trial (NCT03049189), presented at the ENETS 2025 conference, showed significantly longer PFS with [^177^Lu]Lu-DOTA-TOC compared with everolimus in patients with progressive, inoperable, or metastatic SSTR-positive G1-G2 GEP NETs (29.3 vs. 14.1 months, *p* = 0.022). Although OS data remain immature and crossover rates were high, a non-significant trend towards improved OS was observed (63.4 vs. 58.7 months, *p* = 0.206).

Additional phase 3 trials are now evaluating SSTR-targeted RLT in earlier treatment settings and higher-grade GEP NETs. The NETTER-2 trial recently demonstrated that [^177^Lu]Lu-DOTA-TATE significantly prolonged PFS compared with somatostatin analogues alone as first-line therapy in patients with G2-G3 GEP NETs [24], with consistent benefit in all subgroup analyses. The ongoing NETTER-3 trial is assessing [^177^Lu]Lu-DOTA-TATE plus long-acting octreotide versus long-acting octreotide alone as first-line treatment for G1-G2 GEP NETs with high disease burden. The COMPOSE trial (NCT04919226), which has recently completed recruitment, is evaluating [^177^Lu]Lu-DOTA-TOC compared to best standard of care (CAPTEM, everolimus, FOLFOX) in first- or second-line treatment of advanced G2-G3 GEP NETs, with results anticipated soon. Evidence from these studies is expected to support regulatory approval of SSTR-targeted RLT in G3 GEP NETs and in earlier lines of treatment.

Table 2 summarises key randomised phase 3 clinical trials on SSTR-targeted RLT in NETs.

The introduction of SSTR-targeted RLT has significantly advanced the management of neuroendocrine tumours in the past years, providing an effective treatment for a rare disease with historically limited therapeutic options. In addition to improving disease control and survival outcomes, SSTR-targeted RLT plays an important role in symptom relief, particularly in patients with functioning NETs [25]. The treatment is generally well-tolerated, including in elderly patients [26]. Large observational studies have confirmed a favourable safety profile compared to other systemic therapies, with the most commonly reported toxicities being renal and haematological, usually mild, preventable with appropriate precautions, and reversible [27,28].

Ongoing clinical trials on SSTR-targeted RLT are focusing on defining optimal treatment timing and sequencing, individualising therapy through dosimetry, and evaluating strategies such as retreatment, combination therapy, and modified administration protocols. Notably, the phase 3 START-NET trial (NCT05387603) is investigating the benefit of personalised SSTR-targeted RLT by comparing standard treatment with [^177^Lu]Lu-DOTA-TOC for four cycles versus individualised therapy based on kidney dosimetry.

Further areas of active research include the use of SSTR-targeted radiopharmaceuticals labelled with α or Auger electrons emitters, intraarterial administration, and the use of novel ligands such as SSTR antagonists. Among these, the α-emitting agent [^225^Ac]Ac-DOTA-TATE is currently being evaluated in the phase 3 ACTION-1 study (NCT05477576), which is enrolling patients with advanced G1-G2 GEP NETs progressing after ^177^Lu-labelled SSTR ligands.

Moreover, SSTR-targeted RLT is being evaluated in other tumour types that express somatostatin receptors, including lung neuroendocrine neoplasms, neuroblastoma, pheochromocytoma, paraganglioma, meningioma, prostate cancer with neuroendocrine differentiation, and selected subtypes of breast and thyroid cancers.

Supplementary Table S1 summarises key phase 2 trials investigating SSTR-targeted RLT in non-GEP NET malignancies.

### 3.2. PSMA-Targeted Radioligand Therapy

PSMA-targeted RLT agents bind to PSMA, a transmembrane glycoprotein highly expressed on prostate cancer cells and, to a lesser extent, on endothelial cells within the neovasculature of several other tumour types, including hepatocellular carcinoma, renal cancer, thyroid cancer, and glioblastoma [29].

The first PSMA-targeted radiopharmaceutical to receive regulatory approval was [^177^Lu]Lu-PSMA-617 (Pluvicto^®^), a radiolabelled small molecule. [^177^Lu]Lu-PSMA-617 was approved by the FDA in March 2022 and by the EMA in December 2022 for the treatment of PSMA-positive metastatic castration-resistant prostate cancer (mCRPC) following progression on at least one androgen receptor pathway inhibitor (ARPI) and one taxane-based chemotherapy. This was followed by national authorisations in several countries and a rapid adoption into clinical practice and treatment guidelines worldwide. In March 2025, the FDA expanded the indication to include adults with PSMA-positive mCRPC who have been treated with ARPI therapy, and it is considered appropriate to delay taxane-based chemotherapy.

Regulatory approval was initially based on the VISION trial, a randomised, multicentre phase 3 study comparing [^177^Lu]Lu-PSMA-617 plus protocol-permitted standard of care versus standard of care alone. The VISION trial, published in June 2021, demonstrated a significant improvement in radiographic PFS (8.7 vs. 3.4 months, *p* < 0.001) and OS (15.3 vs. 11.3 months, *p* < 0.001) in favour of the [^177^Lu]Lu-PSMA-617 arm [30]. Additional supporting evidence came from the earlier TheraP trial, a phase 2 randomised study published in February 2021, which showed higher PSA response rates and improved PFS in mCRPC patients treated with [^177^Lu]Lu-PSMA-617 versus cabazitaxel [31].

The expanded indication for mCRPC patients progressing after ARPI therapy was supported by the phase 3 PSMAfore trial, published in September 2024, which demonstrated longer median radiographic PFS with [^177^Lu]Lu-PSMA-617 compared to ARPI switch (9.3 vs. 5.55 months, *p* < 0.0001) [32]. The final OS analysis showed no statistically significant difference in median OS (24.5 vs. 23.1 months, *p* = 0.20), although interpretation is impacted by the high rate of crossover from the control to the [^177^Lu]Lu-PSMA-617 arm [33].

Several recently published and ongoing trials are exploring PSMA-targeted RLT in earlier stages of prostate cancer and combination strategies. The ENZA-p trial, a phase 2 randomised study, evaluated [^177^Lu]Lu-PSMA-617 combined with enzalutamide versus enzalutamide alone in first-line mCRPC, showing longer PSA PFS (13 vs. 7.8 months, *p* < 0.0001) [34] and improved OS (34 vs. 26 months, *p* = 0.0053) [35] with the combination. The phase 2 UpFrontPSMA trial assessed [^177^Lu]Lu-PSMA-617 prior to docetaxel in patients with de novo metastatic hormone-sensitive prostate cancer (mHSPC), reporting longer PSA PFS (31 vs. 20 months, *p* = 0.039) and delayed progression to castration-resistant disease (20 vs. 16 months, *p* = 0.033) compared to docetaxel alone [36].

Ongoing phase 3 trials, including PSMAddition (NCT04720157), STAMPEDE2 (NCT06320067), and PEACE6-Poor Responders (NCT06496581), are expected to further clarify the role of [^177^Lu]Lu-PSMA-617 in mHSPC.

In parallel, other phase 3 trials are exploring additional PSMA-targeted agents in patients with mCRPC progressing after at least one ARPI. These include the SPLASH (NCT04647526) and ECLIPSE (NCT05204927) studies, both investigating [^177^Lu]Lu-PSMA-I&T, a radiolabelled small molecule, and the ProstACT GLOBAL studies (NCT06520345, NCT04876651), evaluating [^177^Lu]Lu-DOTA-rosopatamab, a radiolabelled antibody directed against the extracellular domain of PSMA. Table 3 summarises key completed and ongoing phase 3 trials on PSMA-targeted RLT.

PSMA-targeted RLT is reshaping the treatment landscape for prostate cancer, expanding treatment options for patients with advanced prostate cancer and contributing to improved outcomes and quality of life, both in clinical trials and in routine practice [37]. Similar to SSTR-targeted RLT, the treatment is generally well tolerated, including in elderly and frail patients [38]. The most frequently reported toxicities are haematological and renal, with xerostomia also commonly observed due to physiological PSMA uptake in the salivary glands [39,40,41].

Numerous ongoing clinical trials are assessing PSMA-targeted RLT in earlier disease stages, combination regimens strategies, personalised activity regimens dosing strategies, and next-generation radiopharmaceuticals with higher PSMA affinity or alternative radionuclides, including α or Auger electron emitters. Among these, α-emitting agents are expected to represent a major potential breakthrough, based on promising results from the large multicentre retrospective WARMTH Act study and additional early clinical data from smaller cohorts [42,43].

Two multicentre, randomised phase 2/3 trials, AlphaBreak (NCT06402331) and PSMAcTION (NCT06780670), are currently evaluating [^225^Ac]Ac-PSMA-I&T and [^225^Ac]Ac-PSMA-617, respectively, in patients with mCRPC progressing after treatment with ^177^Lu-labelled PSMA radiopharmaceuticals. Moreover, an upcoming phase 3 trial, AcTFirst (NCT06855277), will assess the combination of [^225^Ac]Ac-PSMA-617 and an ARPI in chemotherapy-naïve mCRPC patients who have progressed after one prior ARPI.

Finally, given the presence of PSMA on the neovasculature of other tumour types, including salivary gland tumours, renal cancer, hepatocellular carcinoma, and gliomas, there are ongoing clinical trials investigating the role of PSMA-targeted RLT in non-prostate malignancies, which is summarised in Supplementary Table S2. Its potential role in non-prostate tumours remains to be defined [44].

### 3.3. FAP-Targeted Radioligand Therapy

Fibroblast Activation Protein (FAP)-targeted RLT agents bind FAP, a transmembrane serine protease selectively expressed on cancer-associated fibroblasts, which are present in the tumour microenvironment of approximately 90% of solid tumours. In addition to its expression on stromal cells, FAP has also been detected on the surface of tumour cells in selected malignancies, including pancreatic ductal adenocarcinoma, sarcoma, colorectal cancer, and gastric cancer [45].

The high expression of FAP in multiple solid tumours, combined with minimal expression in normal tissues, has generated strong interest in therapeutic strategies targeting this molecule, including RLT.

To date, no FAP-targeted RLT agent has received regulatory approval, and clinical evidence remains limited. Most available data derive from case reports and retrospective studies. However, preliminary clinical experience with radiolabelled FAP inhibitors has shown encouraging safety and antitumour activity, leading to the initiation of multiple early-phase prospective trials [46].

Most FAP-targeted agents evaluated so far are small molecules or peptides, including FAPI-46 and FAP-2286, labelled with β emitters including lutetium-177. More recently, FAP-targeting agents labelled with the α emitter actinium-225 have also been explored, with initial results presented at the 2025 SNMMI Annual Meeting in patients with metastatic colorectal cancer [47]. The tumour types most frequently investigated in early clinical studies include head and neck cancers, particularly thyroid cancer, breast cancer, pancreatic cancer, colorectal cancer, and sarcoma. Initial prospective evidence came from a phase 1 study (NCT05410821), published in December 2023, which evaluated [^177^Lu]Lu-DOTA-EB-FAPI, an albumin-binding small molecule, in 12 patients with radioactive iodine-refractory thyroid cancer. The study reported an ORR of 25% and a disease control rate (DCR) of 83%, with grade 3–4 haematological toxicity observed in 17% of patients treated at the highest administered activity of 4.99 GBq per cycle during dose escalation [48].

A subsequent phase 2 trial, published in February 2025, investigated the same radiopharmaceutical in 28 patients with refractory or progressive, heavily pretreated solid tumours. This study demonstrated an ORR of 20% and a DCR of 65%, with grade 3–4 haematological toxicity reported in 21% of patients receiving 3.3 GBq/cycle every 6 weeks [49].

By contrast, the FRONTIER phase 1 trial (NCT05432193), which evaluated [^177^Lu]Lu-PNT6555 in patients with advanced or metastatic solid tumours, including pancreatic adenocarcinoma, high-grade soft tissue sarcoma, oesophageal cancer, colorectal cancer, melanoma, head and neck squamous cell carcinoma, and cholangiocarcinoma, was terminated early due to rapid clearance of the agent, resulting in low tumour absorbed doses and limited efficacy [50].

Several additional early-phase trials are currently ongoing, investigating different FAP-targeted agents either as monotherapy or in combination regimens in various solid tumour types. Supplementary Table S3 summarises completed and ongoing clinical trials with FAP-targeting RLT agents.

### 3.4. CAIX-Targeted Radioligand Therapy

CAIX is a transmembrane enzyme involved in pH regulation within the tumour microenvironment, where it promotes cancer cell survival, proliferation, and invasion. Its expression is tightly regulated by hypoxia, primarily through activation of hypoxia-inducible factor 1 alpha (HIF-1α). CAIX localises to the surface of hypoxic tumour regions and is highly and homogeneously expressed in clear cell renal cell carcinoma (ccRCC). In ccRCC, loss-of-function mutations in the von Hippel-Lindau (*VHL*) gene, present in approximately 95% of cases, lead to constitutive activation of HIF-1α and subsequent CAIX upregulation [51].

Over recent years, CAIX has emerged as a promising target for imaging and therapy in hypoxic tumours, particularly ccRCC. In vivo expression has been confirmed in nearly 90% of histologically proven ccRCC using PET/CT with the radiolabelled antibody [^89^Zr]Zr-DFO-girentuximab [52].

Clinical experience with CAIX-targeted RLT is currently limited. Most studies to date have enrolled patients with advanced or metastatic ccRCC, although other indications are being explored. The most extensively studied radiopharmaceutical is [^177^Lu]Lu-DOTA-girentuximab.

Two early-phase trials conducted in the Netherlands evaluated [^177^Lu]Lu-DOTA-girentuximab in ccRCC.

A phase 1 dose-escalation study in 23 patients tested activity levels between 1100 and 2590 MBq/m^2^ and identified a maximum tolerated dose 2405 MBq/m^2^ [53]. Disease control after the first cycle was 78%, but grade 3–4 haematological toxicity occurred in 58% of patients receiving ≥1850 MBq/m^2^ [53]. A subsequent phase 2 study in 14 patients using the maximum tolerated dose reported a DCR of 64% after one cycle, with grade 3–4 haematologic toxicity in 86% of participants [54]. These findings highlight the dose-limiting myelotoxicity of [^177^Lu]Lu-DOTA-girentuximab at higher activity levels.

The ongoing STARSTRUCK trial (NCT05868174) is evaluating [^177^Lu]Lu-DOTA-girentuximab in combination with peposertib, a DNA-dependent protein kinase inhibitor with radiosensitising properties [55]. Notably, this study also includes patients with other solid tumours, provided CAIX expression is confirmed at screening with [^89^Zr]Zr-DFO-girentuximab PET, and adopts lower fixed activities (1887–3145 MBq, approximately corresponding to 1100–1850 MBq/m^2^) to improve tolerability compared with previous protocols.

In parallel, additional CAIX-targeting radiopharmaceuticals are under investigation in preclinical and early phase clinical trials, including radiolabelled small molecules and peptides. A phase 1/2 trial (NCT05706129) is currently assessing [^177^Lu]Lu-DPI-4452, a peptide ligand targeting CAIX [56], in patients with ccRCC as well as pancreatic adenocarcinoma and colorectal cancer.

The therapeutic potential of CAIX-targeted RLT in ccRCC, either as monotherapy or in combination with TKIs or immunotherapy, remains to be determined in larger, late-phase trials. To date, no phase 3 studies have been initiated. Patients with von Hippel-Lindau syndrome may represent another population of interest for future studies, given their predisposition to develop multiple CAIX-expressing tumours, including ccRCC, haemangioblastomas, and neuroendocrine tumours, all driven by *VHL* inactivation [57].

The clinical utility of CAIX-targeted RLT in solid tumours other than ccRCC also remains an open question that future studies will need to address. This uncertainty is partly explained by biological differences in CAIX expression between ccRCC and other malignancies. While expression in ccRCC is typically homogeneous and consistent between primary and metastatic lesions due to *VHL* mutation, expression in other malignancies may be heterogeneous, patchy, and hypoxia dependent.

Supplementary Table S4 summarises ongoing clinical trials of CAIX-targeting RLT agents.

### 3.5. GRPR-Targeted Radioligand Therapy

Gastrin-Releasing Peptide Receptor (GRPR)-targeted RLT agents bind to GRPR, a G-protein-coupled receptor implicated in tumour proliferation, migration, and metastasis. GRPR is expressed on the cell surface in multiple solid tumours [58] and has recently emerged as a promising target for imaging and therapy, particularly in prostate cancer, breast cancer, and glial tumours.

Clinical development of GRPR-targeted RLT is still at an early stage. Several phase 1 and 1/2 trials are currently evaluating the safety and preliminary efficacy of GRPR-targeted radiopharmaceuticals in selected solid tumour types.

The most extensively investigated agent is [^177^Lu]Lu-NeoB, a radiolabelled GRPR-binding peptide. It is under evaluation in a phase 1/2 trial (NCT06247995) in combination with capecitabine, and in a separate phase 1 study in combination with ribociclib and fulvestrant in patients with advanced or metastatic ER-positive, HER2-negative breast cancer. Additional early-phase studies are assessing [^177^Lu]Lu-NeoB in glioblastoma (NCT05739942) and other GRPR-expressing solid tumours (NCT03872778).

[^177^Lu]Lu-NeoB is also being investigated in a personalised treatment approach in the NEPC phase 1 trial (NCT06379217), which enrols patients with metastatic neuroendocrine prostate cancer. Participants undergo PET imaging with [^68^Ga]Ga-NeoB, [^68^Ga]Ga-PSMA-11 and [^68^Ga]Ga-DOTA-TATE and are subsequently treated with the corresponding therapeutic agent according to the target showing the highest expression.

Other GRPR-targeted radiopharmaceuticals are also in development. A phase 1/2 trial (NCT05633160) is evaluating [^67^Cu]Cu-SAR-BBN, a peptide labelled with the β-emitter copper-67, in patients with mCRPC who are ineligible for [^177^Lu]Lu-PSMA therapy. Another study (NCT05283330) is investigating [^212^Pb]Pb-DOTAM-GRPR1, an α-emitting GRPR-targeted radioligand, in patients with metastatic GRPR-positive solid tumours.

Supplementary Table S5 summarises ongoing clinical trials of GRPR-targeted RLT.

### 3.6. Emerging Targets

An increasing number of novel RLT agents are under clinical investigation. Among the most promising emerging targets are chemokine receptor 4 (CXCR4), delta-like ligand 3 (DLL3), folate receptor α (FRα) integrins (αvβ3 and αvβ3), six-transmembrane epithelial antigen of the prostate 2 (STEAP2), insulin-like growth factor 1 receptor (IGF-1R), melanocortin 1 receptor (MC1R), nectin-4, human epidermal growth factor receptor 2 (HER2), and selected cluster of differentiation (CD) markers.

CXCR4, a chemokine G-protein-coupled receptor, is implicated in tumour growth, invasion, angiogenesis, and metastasis. It is highly expressed in several haematological malignancies such as leukaemia and multiple myeloma, as well as in solid tumours including NETs, pancreatic adenocarcinoma, glioblastoma, colorectal cancer, and non-small-cell lung cancer (NSCLC) [59]. CXCR4-targeted radiopharmaceuticals such as [^177^Lu]Lu-PentixaTher and [^212^Pb]Pb-PentixaTher are being investigated in early phase trials for refractory acute leukaemia and lung NETs (NCT06356922, NCT05557708).

DLL3, an inhibitory ligand of the Notch signalling pathway, is overexpressed in high-grade neuroendocrine tumours, particularly small-cell lung cancer, large-cell neuroendocrine carcinoma, and poorly differentiated neuroendocrine carcinoma [60]. DLL3-targeted radioligands, including [^177^Lu]Lu-DTPA-SC16.56 and [^225^Ac]Ac-ETN029, are being evaluated in early-phase studies (NCT06941480, NCT07006727).

FRα is overexpressed in several epithelial malignancies, most notably NSCLC, ovarian, breast, and colorectal cancers [61]. The FRα-targeted agent [^177^Lu]Lu-EVS459 is under investigation in a phase 1 trial enrolling in patients with progressive high-grade serous ovarian cancer or non-squamous NSCLC (NCT06376253).

STEAP2, a membrane protein involved in metal ion transport, contributes to tumour progression in prostate cancer and hepatocellular carcinoma [62,63]. A phase 1 trial is evaluating [^225^Ac]Ac-AZD2284, a radiolabelled antibody targeting STEAP2, in patients with mCRPC (NCT06879041).

IGF-1R is a transmembrane receptor expressed in pancreatic, colorectal, and prostate cancers [64]. The α-emitting agent [^225^Ac]Ac-FPI-1434 is being studied in a phase 1 trial of advanced solid tumours refractory to standard therapy (NCT03746431).

Integrins such as αvβ3 and αvβ6 are cell adhesion receptors involved in tumour proliferation and invasion and are frequently upregulated in solid tumours [65]. Radiolabelled peptides targeting these integrins are being tested in early-phase studies in breast cancer, NSCLC, pancreatic adenocarcinoma, and other tumour types (NCT06375564, NCT05013086, NCT06389123, NCT06228482, NCT04665947).

HER2, a receptor tyrosine kinase, is overexpressed in breast and gastric cancer, and less commonly in other epithelial malignancies [66]. Several trials are evaluating HER2-directed radiolabelled antibodies in patients with HER2-positive solid tumours (NCT06824155, NCT05982626).

MC1R is a receptor frequently overexpressed in melanoma. α-emitting MC1R-targeted peptides are being investigated in early-phase trials in patients with advanced or metastatic cutaneous or uveal melanoma, including in combination with immunotherapy (NCT05655312, NCT05496686).

Nectin-4, a cell adhesion molecule involved in tumour growth, migration, and angiogenesis, is overexpressed is several epithelial malignancies. The phase 1 NECTINIUM-2 trial (NCT07020117) is currently evaluating [^225^Ac]Ac-AKY-1189, a radiolabelled mini-protein targeting nectin-4, in patients with metastatic solid tumours.

In addition, several radiolabelled monoclonal antibodies directed against CD markers are under development, particularly in haematological malignancies.

Finally, bispecific radiopharmaceuticals have recently emerged as a strategy to enhance tumour targeting by simultaneously binding two molecular targets. Early-phase trials are evaluating [^225^Ac]Ac-FPI-2068, a bispecific antibody targeting EGFR/c-MET (NCT06147037), [^177^Lu]Lu-DOTA-FAPI-RGD, a bispecific peptide binding both FAP and integrin αvβ3 (NCT06638034), and [^177^Lu]Lu-TATE-RGD, a bispecific peptide targeting SSTR2 and integrin αvβ3 (NCT06632873).

## 4. Global Access and Availability of Radioligand Therapy

Despite growing clinical evidence and regulatory approvals, the global expansion of RLT is limited by marked disparities in access and availability. The Radiotherapy and Theranostics Lancet Oncology Commission recently reported wide variability in the use of SSTR- and PSMA-targeted therapeutic radiopharmaceuticals based on national income levels [67]. The global average use of ^177^Lu-labelled SSTR-targeted peptides was 3.1 per million population, with high-income countries averaging 16.3 per million, compared with 1.2 per million in upper-middle-income and 0.6 per million in lower-middle-income countries. No use was reported in low-income countries, including most of Asia, Latin America, and Africa.

A similar pattern was observed for PSMA-targeted RLT. The global average use of ^177^Lu-labelled PSMA-targeted radiopharmaceuticals was 2.7 per million population, with high-income countries reporting 14.2 per million, compared with 1.5 per million in upper-middle-income countries and 0.3 per million in lower-middle-income countries. As with SSTR-targeted agents, no use was documented in low-income countries.

A regional survey of theranostic capacity in 15 West Asian countries reported availability of SSTR- and PSMA-targeted RLT in 12 countries, with no access in Yemen, Palestine, and Syria [68]. Access to newer agents, including FAP- and CXCR4-targeted radiopharmaceuticals, was even more restricted and confined to Turkey, Iran, Azerbaijan, and Oman. Overall, these data reflect current inequalities in RLT availability and highlight the need for coordinated efforts to expand access.

Workforce shortages represent a major barrier to the equitable implementation of RLT. A global survey conducted by the Lancet Oncology Commission in 189 countries showed that many low- and middle-income countries lack sufficient numbers of trained nuclear medicine physicians, radiopharmacists, radiochemists, and technologists [69]. Even in high-income countries, workforce capacity is often insufficient to meet current or projected demand. The growing number of approved indications, regulatory approvals of new radiopharmaceuticals, and rapid integration of theranostics into oncology practice are expected to exacerbate these shortages.

Educational gaps further limit the development of a sustainable workforce. In many countries, national training programmes in nuclear medicine do not include structured curricula for RLT, and few offer dedicated specialisation pathways in theranostics [69]. Revision and harmonisation of training standards are urgently needed. The International Atomic Energy Agency (IAEA) and professional organisation such as the World Federation of Nuclear Medicine and Biology (WFNMB), European Association of Nuclear Medicine (EANM), Australasian Association of Nuclear Medicine Specialists (AANMS), and the Society of Nuclear Medicine and Molecular Imaging (SNMMI) are working to establish educational frameworks and programs to ensure an adequate number of trained professionals in theranostics to meet current and future clinical demand.

Infrastructure limitations represent another major challenge. Radiopharmaceutical production relies on a limited number of high-capacity reactors, most of which are located in high-income countries [70]. Many low- and middle-income countries lack domestic facilities for radiopharmaceutical manufacturing, as well as appropriate regulatory frameworks, handling infrastructure, and radioactive waste management systems. Where local production is not available, access depends on international supply chains, which are often limited by high costs, transport regulations, and the short radiopharmaceutical shelf-life. Notably, lutetium-177, the most commonly used radionuclide in RLT, is produced by a limited number of reactors, with current global capacity sufficient to treat fewer than 200,000 patients annually [70]. By contrast, a modelling analysis by the Radiotherapy and Theranostics Lancet Oncology Commission (performed before the recent FDA label expansion for [^177^Lu]Lu-PSMA-617) estimated that more than 158,000 patients could be eligible for PSMA RLT annually, requiring over 550,000 treatment cycles worldwide [67]. This estimate accounts only for [^177^Lu]Lu-PSMA-617 and does not include patients treated with [^177^Lu]Lu-DOTA-TATE or other investigational ^177^Lu-based therapies. The comparison highlights the significant imbalance between current capacity and projected global demand. Expanded reactor capacity, alternative production technologies, and decentralised manufacturing will be essential to bridge this gap.

Regulatory variability and funding gaps further hinder access. Reimbursement of therapeutic radiopharmaceuticals and companion imaging is inconsistent, and many health systems lack dedicated reimbursement pathways [70]. Approval processes also vary widely between countries, even for agents authorised by the EMA or FDA, creating further delays in access. The infrastructure and workforce investments required to support RLT expansion amplify the challenge, particularly in low- and middle-income countries. Addressing these deficits in production, workforce, infrastructure, and funding will be fundamental to meet clinical demand and ensure equitable global access as the field continues to grow.

## 5. Optimising Radioligand Therapy: Current Clinical Challenges

The approval of [^177^Lu]Lu-DOTA-TATE and [^177^Lu]Lu-PSMA-617 marked a turning point in the field of RLT, drawing attention to its therapeutic potential and laying the foundation for wider clinical adoption. However, as a relatively new therapeutic modality, RLT is evolving rapidly, and numerous challenges and unanswered questions must be addressed to optimise its use and expand its role in oncology [66,71].

A major limitation of currently approved agents is the use of fixed dosing regimens, in which the administered activity (GBq) is standardised, but the radiation absorbed dose (Gy) delivered to tumours and normal organs may vary markedly between patients. Because absorbed dose depends on individual pharmacokinetics and tumour characteristics, identical administered activities can result in wide interpatient variability in radiation absorbed doses. For [^177^Lu]Lu-PSMA-617 and [^177^Lu]Lu-DOTA-TATE, approximately half of patients are estimated to be either under or overtreated with the standard protocols (intravenous administration of 7.4 GBq every six weeks for [^177^Lu]Lu-PSMA-617, or every eight weeks for [^177^Lu]Lu-DOTA-TATE) [72], and about two-thirds of the cumulative tumour absorbed dose is delivered within the first two cycles [73]. These regimens do not consider important factors such as tumour burden, level of target expression based on pre-treatment imaging, intra- and interlesional heterogeneity in target expression, tumour radiosensitivity, and baseline organ function. Theoretically, kidney and bone marrow absorbed doses should remain below the conventional limits established for external beam radiotherapy (23 Gy and 2 Gy, respectively) to minimise the risk of nephrotoxicity and haematologic toxicity, while tumour absorbed doses should ideally range between 100 and 200 Gy to achieve therapeutic efficacy [74,75,76,77]. Several retrospective and small prospective studies have supported the relevance of the absorbed dose-effect relationship [78,79,80,81,82], which directly links tumour response and toxicity risk to the absorbed dose, and have suggested potential benefits of dosimetry-guided therapy [83,84,85]. Building on this preliminary evidence, many ongoing clinical trials now include pre- and post-treatment dosimetry to tailor administered activity (NCT06943495, NCT05896371, NCT04917484). Although biologically compelling, dosimetry-guided therapy has not yet demonstrated superiority over fixed-activity regimens in prospective randomised trials [86]. Even if future studies confirm a clinical benefit, widespread implementation is likely to be challenging due to practical limitations, including supply chain and infrastructural constraints, the complexity of individualised dose planning, and dose-limiting toxicities such as xerostomia in PSMA-targeted RLT. Optimisation of treatment intervals and de-escalation strategies in selected cases are also being evaluated (NCT03454763, NCT06216249, NCT06200103).

Another important clinical challenge is the lack of response or the development of resistance during therapy. Despite confirmation of target expression on baseline imaging, a subset of patients either fails to respond or relapse after an initial response. The biological mechanisms underlying resistance are poorly understood, and several studies are investigating these pathways [87,88,89] (NCT05435495). Strategies currently under evaluation include dose escalation in patients with low baseline target expression (NCT06526299), combination with agents that enhance target expression (such as ARPIs for PSMA or somatostatin analogues for SSTR), and combination with radiosensitisers that enhance the cytotoxic effect of radiation by interfering with DNA repair. Alternative routes of administration are also under evaluation, including intra-arterial delivery for patients with liver-dominant metastatic neuroendocrine tumours (NCT03590119) or central nervous system tumours such as meningiomas and gliomas.

Toxicity is also a relevant concern, especially haematologic and renal toxicity. Although long-term safety data support the feasibility of extending treatment beyond the standard four or six cycles [90,91], a proportion of patients discontinue therapy due to adverse events. Notably, bone marrow toxicity is more closely related to the absorbed dose than to the number of treatment cycles and may be prevented through maintaining the biologically effective dose to the bone marrow below 2 Gy [92]. Ongoing trials are evaluating the safety of RLT in patients with baseline renal impairment or cytopaenias secondary to bone marrow involvement (NCT06004661, NCT07025512), as well as the feasibility of retreatment following completion of standard therapy (NCT06866938, NCT06288113, NCT05773274, NCT04954820).

Patient selection and response assessment are additional areas of active investigation. Currently, there is substantial variability and no international consensus on how patients should be selected for RLT or how treatment response should be assessed [93]. For example, in the United States, eligibility for [^177^Lu]Lu-PSMA-617 typically relies on baseline PSMA PET, whereas in Australia, dual-tracer imaging with PSMA and FDG PET is commonly used to exclude patients with discordant PSMA-negative/FDG-positive disease, a poor prognosis subgroup unlikely to benefit from [^177^Lu]Lu-PSMA-617. Although certain clinical and imaging features, including multiple prior lines of therapy, impaired baseline organ function, low target expression, and FDG-avid dedifferentiated disease have been associated with reduced benefit [94,95,96,97], the ability to reliably predict treatment response remains limited. Improved clinical, genomic, and imaging biomarkers are needed to refine eligibility, identify patients most likely to benefit, and avoid ineffective treatment. In this context, emerging approaches such as artificial intelligence-based models may provide clinical decision support tools to improve the prediction of absorbed dose to tumours and normal organs in individual patients, enabling more accurate patient selection and treatment planning [98,99].

An equally important challenge is response evaluation. For both [^177^Lu]Lu-DOTA-TATE and [^177^Lu]Lu-PSMA-617, response assessment is inconsistent across centres [93]. No universally recognised imaging-based criteria currently exist, and response evaluation is often based on a combination of imaging, tumour markers, and clinical endpoints. To address this gap, the Response Evaluation Criteria in PSMA PET/CT (RECIP 1.0) were recently proposed [100]. These criteria use visual or quantitative assessment of total tumour volume and the identification of new lesions on PSMA PET and have demonstrated good prognostic value and interreader agreement [101]. Similar efforts are needed to establish standardised response criteria for SSTR-targeted RLT [102]. Consensus for terminology for use in describing and reporting results of theranostic trials has also been reported [103].

As the field advances, personalised treatment schedules, refined patient selection, and standardised response assessment will be essential to optimise RLT.

## 6. Conclusions

RLT is emerging as one of the most promising targeted treatment approaches in oncology, with more than 400 clinical trials registered as of July 2025. Two radiopharmaceuticals, [^177^Lu]Lu-DOTA-TATE (Lutathera^®^) and [^177^Lu]Lu-PSMA-617 (Pluvicto^®^), are now approved for advanced neuroendocrine tumours and prostate cancer, and several others are in development for both solid and haematologic malignancies. Ongoing research is focusing on new molecular targets such as FAP, CAIX, and GRPR, as well as on novel radionuclides and combination regimens.

Clinical adoption, however, continues to be restricted by uneven global access, shortages in radionuclide production and workforce capacity, and fragmented regulatory pathways. Current practice is further limited by fixed treatment schedules, despite growing evidence supporting personalised approaches guided by tumour burden, target expression, and dosimetry. Resistance, heterogeneous responses, and toxicity highlight the need for better patient selection, more personalised treatment strategies, and a deeper understanding of the radiobiological mechanisms involved.

In the coming years, RLT is expected to expand its indications, move into earlier treatment lines, and be rationally combined with other systemic and local therapies. Progress will also depend on optimising radiopharmaceutical design, through the development of ligands with improved tumour retention, isotope selection tailored to disease and patient characteristics, and advances in radiochemistry to improve stability, selectivity, and ultimately efficacy and safety.

With continued investment in research, infrastructure, and workforce capacity, RLT has the potential to evolve from an emerging and highly specialised treatment modality into an integral component of routine oncology practice and precision cancer care.

## Figures and Tables

**Table 1:** Radioligand therapy agents approved by the FDA and EMA for clinical use.

	[^177^Lu]Lu-DOTA-TATE (Lutathera^®^)	[^177^Lu]Lu-PSMA-617 (Pluvicto ^®^)
**Target**	SSTR2	PSMA
**Ligand**	TATE (peptide)	PSMA-617 (small molecule)
**Linker**	DOTA (non-cleavable)	DOTA (non-cleavable)
**Radionuclide**	Lutetium-177 (β-emitter)	Lutetium-177 (β-emitter)
**Year of FDA approval**	2018	2022
**Year of EMA approval**	2017	2022
**Approved indication(s)**	Progressive, unresectable or metastatic, SSTR-positive G1-G2 GEP NETs in adults (FDA, EMA) Progressive, unresectable or metastatic, SSTR-positive G1-G2 GEP NETs in paediatric patients ≥ 12 years (FDA)	PSMA-positive mCRPC previously treated with ARPI and taxane-based CT (FDA, EMA) PSMA-positive mCRPC previously treated with ARPI and considered appropriate to delay taxane-based chemotherapy (FDA)
**Standard activity administration**	7.4 GBq (200 mCi) IV every 8 weeks for 4 cycles; co-infusion of amino acids for renal protection	7.4 GBq (200 mCi) IV every 6 weeks for up to 6 cycles
**Common adverse effects**	Fatigue, nausea, vomiting, diarrhea, abdominal pain, myelosuppression, (anaemia, thrombocytopaenia, lymphocytopaenia), nephrotoxicity	Fatigue, nausea, vomiting, diarrhea, myelosuppression (anaemia, thrombocytopaenia, lymphocytopaenia), nephrotoxicity, xerostomia, dry eyes

CT: chemotherapy; EMA: European Medicines Agency; FDA: U.S. Food and Drug Administration; IV: intravenous; mCRPC: metastatic castration-resistant prostate cancer; PSMA: prostate specific membrane antigen; SSTR2: somatostatin receptor type 2.

**Table 2:** Summary of key completed and ongoing randomised phase 3 trials of SSTR-targeted RLT in neuroendocrine tumours.

NCT Number	Completion, y	Radiopharmaceutical	Patients, n	Patient Population	Combination	Comparator Arm	Sponsor	Outcome Data
NCT01578239 (NETTER-1)	2021	[^177^Lu]Lu-DOTA-TATE	231	Progressive, inoperable or metastatic SSTR+ G1-G2 midgut NETs	Octreotide LAR	Octreotide LAR	Advanced Accelerator Applications *	mPFS: 28.4 vs. 8.4 mo (*p* < 0.001) mOS: 48 mo vs. 36.3 mo (*p* = 0.30)
NCT03972488 (NETTER-2)	2027	[^177^Lu]Lu-DOTA-TATE	226	Treatment-naïve inoperable or metastatic G2-G3 GEP NETs	Octreotide LAR	Octreotide LAR	Advanced Accelerator Applications *	mPFS: 22.8 vs. 8.5 (*p* < 0.001)
NCT04919226 (COMPOSE)	2027	[^177^Lu]Lu-DOTA-TOC	259	Advanced G2-G3 GEP NETs (first or second line of treatment)	None	Best SoC	ITM Solucin GmbH	NA
NCT03049189 (COMPETE)	2029	[^177^Lu]Lu-DOTA-TOC	309	Progressive, inoperable or metastatic SSTR+ G1-G2 GEP NETs	None	Everolimus	ITM Solucin GmbH	mPFS: 29.3 vs. 14.1 mo (*p* = 0.022) ^†^ mOS: 63.4 vs. 58.7 mo (*p* = 0.206) ^†^
NCT06784752 (NETTER-3)	2034	[^177^Lu]Lu-DOTA-TATE	240	Newly diagnosed metastatic or locally advanced G1-G2 GEP NET	Octreotide LAR	Octreotide LAR	Novartis Pharmaceuticals	NA

* Advanced Accelerator Applications was acquired by Novartis in 2018. ^†^ Data from interim conference presentations. GEP: gastroenteropancreatic; LAR: long-acting release; mOS: median overall survival; mPFS: median progression-free survival; NA: not available; NET: neuroendocrine tumour; SoC: standard of care; SSTR: somatostatin receptor.

**Table 3:** Summary of key completed and ongoing randomised phase 3 trials of PSMA-targeted RLT in prostate cancer.

**NCT Number**	Completion, y	Radiopharmaceutical	Patients, n	Patient Population	Combination	Comparator Arm	Sponsor	Outcome Data
NCT03511664 (VISION)	2023	[^177^Lu]Lu-PSMA-617	831	mCRPC post ARPI, post taxane CT	SoC	SoC	Endocyte	mPFS: 8.7 vs. 3.4 mo (*p* < 0.001) mOS: 15.3 vs. 11.3 mo (*p* < 0.001)
NCT04689828 (PSMAfore)	2025	[^177^Lu]Lu-PSMA-617	470	mCRPC post ARPI	None	ARPI change	Novartis Pharmaceuticals	mPFS: 11.6 vs. 5.6 mo (*p* < 0.001) mOS: 24.5 vs. 23.1 mo (*p* = 0.20)
NCT04720157 (PSMAddition)	2026	[^177^Lu]Lu-PSMA-617	1145	mHSPC	SoC (ARPI + ADT)	SoC (ARPI + ADT)	Novartis Pharmaceuticals	NA
NCT04647526 (SPLASH)	2028	[^177^Lu]Lu-PSMA-I&T	415	mCRPC post ARPI	None	ARPI change	POINT Biopharma	mPFS: 9.5 vs. 6.0 mo (*p* = 0.0088) * mOS: 20.8 vs. NE mo (*p* = 0.615) *
NCT04876651 (ProstACT GLOBAL)	2028	[^177^Lu]Lu-DOTA- rosopatamab	392	mCRPC post ARPI	SoC	SoC	Telix Pharmaceuticals	NA
NCT05204927 (ECLIPSE)	2029	[^177^Lu]Lu-PSMA-I&T	439	mCRPC post ARPI	None	ARPI change	Curium US LLC	NA
NCT05939414 (PSMA-DC)	2030	[^177^Lu]Lu-PSMA-617	450	OMPC progressing after primary treatment	SBRT	SBRT	Novartis Pharmaceuticals	NA
NCT06520345 (ProstACT GLOBAL)	2030	[^177^Lu]Lu-DOTA- rosopatamab	430	mCRPC post ARPI	SoC	SoC	Telix Pharmaceuticals	NA
NCT06320067 (STAMPEDE2)	2034	[^177^Lu]Lu-PSMA-617	1756	mHSPC	SoC	SoC	University College, London	NA
NCT06496581 (PEACE6-Poor Responders)	2039	[^177^Lu]Lu-PSMA-617	500	mHSPC	SoC	SoC	UNICANCER	NA

* Data from interim conference presentations. ARPI: androgen receptor pathway inhibitor; CT: chemotherapy; mCRPC: metastatic castration-resistant prostate cancer; mHSPC: metastatic hormone-sensitive prostate cancer; mOS: median overall survival; mPFS: median progression-free survival; NA: not available; NE: non-estimable; OMPC: oligometastatic prostate cancer; PSMA: prostate-specific membrane antigen; SBRT: stereotactic body radiation therapy; SoC: standard of care.

## Data Availability

No new data were created or analyzed in this study.

## Supplementary Materials

The following supporting information can be downloaded at: https://www.mdpi.com/article/10.3390/cancers17213412/s1, Table S1: Key phase 2 and 3 clinical trials investigating SSTR-targeted RLT in non-GEP neuroendocrine and other malignancies registered on ClinicalTrials.gov; Table S2: Key phase 2 and 3 clinical trials investigating PSMA-targeted RLT in non-prostate malignancies registered on ClinicalTrials.gov; Table S3: Clinical trials of FAP-targeted RLT agents registered on ClinicalTrials.gov; Table S4: Clinical trials of CAIX-targeted RLT agents registered on ClinicalTrials.gov; Table S5: Clinical trials of GRPR-targeted RLT agents registered on ClinicalTrials.gov.

## Abbreviations

The following abbreviations are used in this manuscript:

ARPI	Androgen receptor pathway inhibitor
CAIX	Carbonic anhydrase IX
ccRCC	Clear cell renal cell carcinoma
CD	Cluster of differentiation
CXCR4	C-X-C chemokine receptor type 4
DCR	Disease control rate
DLL3	Delta-like ligand 3
EANM	European Association of Nuclear Medicine
EMA	European Medicines Agency
FAP	Fibroblast activation protein
FDA	U.S. Food and Drug Administration
FRα	Folate receptor alpha
GEP	Gastroenteropancreatic
GRPR	Gastrin-releasing peptide receptor
HER2	Human epidermal growth factor receptor 2
IAEA	International Atomic Energy Agency
IGF-1R	Insulin-like growth factor 1 receptor
LET	Linear energy transfer
MC1R	Melanocortin 1 receptor
mCRPC	Metastatic castration-resistant prostate cancer
mHSPC	Metastatic hormone-sensitive prostate cancer
NET	Neuroendocrine tumour
NSCLC	Non-small-cell lung cancer
ORR	Objective response rate
OS	Overall survival
PET	Positron emission tomography
PFS	Progression-free survival
PSMA	Prostate-specific membrane antigen
ROS	Reactive oxygen species
RLT	Radioligand therapy
SNMMI	Society of Nuclear Medicine and Molecular Imaging
SSTR	Somatostatin receptor
STEAP2	Six-transmembrane epithelial antigen of the prostate 2
TKI	Tyrosine kinase inhibitor
