# Supplementary material for: Radioligand Therapy in Cancer Management: A Global Perspective
Source: Cancers (Basel). 2025 Oct 23;17(21):3412. doi: 10.3390/cancers17213412 (PMC12607575; doi:10.3390/cancers17213412)
Supplement: Supplementary file 1 [file cancers-17-03412-s001.zip › cancers-3917891-supplementary.pdf]

**Search strategy**

A search was performed on ClinicalTrials.gov on 5 July 2025 using the keywords radioligand therapy OR RLT OR targeted radionuclide therapy OR peptide receptor radionuclide therapy OR PRRT OR PSMA OR SSTR OR DOTATATE OR FAP OR CAIX OR GRPR OR CXCR4 OR 177Lu OR 131I OR 67Cu OR 47Sc OR 225Ac OR 212Pb OR 211At OR 227Th OR 161Tb OR 90Y. Only interventional studies involving radioligand therapy agents were included. Observational studies and diagnostic-only imaging trials were excluded.

**Supplementary Table S1.** Key phase 2 and 3 clinical trials investigating SSTR-targeted RLT in non-GEP neuroendocrine and other malignancies registered on ClinicalTrials.gov.

| NCT Number | Estimated Completion, y | Phase | Radiopharmaceutical | Patients, n | Patient Population | Combination | Comparator Arm | Sponsor |
|---|---|---|---|---|---|---|---|---|
| NCT05918302 (LEVEL) | 2028 | 3 (randomised) | [$^{177}$Lu]Lu-DOTATOC | 120 | Advanced or metastatic lung or thymic NETs | None | Everolimus | Grupo Espanol de Tumores Neuroendocrinos |
| NCT05691465 | 2025 | 2 | [$^{177}$Lu]Lu-DOTATATE | 30 | Metastatic prostate cancer with neuroendocrine differentiation | None | None | National Cancer Institute (NCI) |
| NCT04665739 | 2025 | 2 (randomised) | [$^{177}$Lu]Lu-DOTATATE | 70 | Recurrent or advanced, well or moderately differentiated lung NETs | None | Everolimus | National Cancer Institute (NCI) |
| NCT05198479 | 2025 | 2 | [$^{177}$Lu]Lu-DOTATATE | 25 | Metastatic, SSTR+ nasopharyngeal carcinoma | None | None | National Cancer Centre, Singapore |
| NCT04529044 | 2025 | 2 | [$^{177}$Lu]Lu-DOTATATE | 10 | Metastatic breast cancer refractory to standard treatment | None | None | OHSU Knight Cancer Institute |
| NCT05583708 | 2026 | 2 | [$^{177}$Lu]Lu-DOTATATE | 18 | Advanced or metastatic Merkel cell carcinoma | Pembrolizumab | None | Weill Medical College of Cornell University |
| NCT06121271 | 2027 | 2 | [$^{177}$Lu]Lu-DOTATATE | 110 | Advanced or metastatic bronchial or thymic NETs, paraganglioma, pheochromocytoma, MTC | None | None | University College, London |
| NCT06045260 (LUFOR) | 2027 | 2 | [$^{177}$Lu]Lu-DOTATATE | 100 | Advanced or metastatic solid tumours refractory to standard treatment | None | None | Istituto Romagnolo per lo Studio dei Tumori Dino Amadori IRST S.r.l. IRCCS |

| | | | | | | | | |
|---|---|---|---|---|---|---|---|---|
| NCT03206060 | 2027 | 2 | [$^{177}$Lu]Lu-DOTATATE | 130 | Advanced pheochromocytoma and paraganglioma | None | None | National Cancer Institute (NCI) |
| NCT03971461 | 2027 | 2 | [$^{177}$Lu]Lu-DOTATATE | 32 | Residual or recurrent grade 1-3 meningioma | None | None | NYU Langone Health |
| NCT06326190 (LUMEN-1) | 2028 | 2 (randomised) | [$^{177}$Lu]Lu-DOTATATE | 135 | Refractory grade 1-3 meningioma | None | Local SoC | European Organisation for Research and Treatment of Cancer - EORTC |
| NCT06126588 (ELUMEN) | 2028 | 2 | [$^{177}$Lu]Lu-DOTATATE | 28 | Refractory grade 2-3 meningioma | Everolimus | None | Central Hospital, Nancy, France |
| NCT06607692 (LuPARPed) | 2029 | 2 | [$^{177}$Lu]Lu-DOTATATE | 25 | Relapsed or refractory SSTR+ paediatric solid tumours | Olaparib | None | Fundación de investigación HM |
| NCT06955169 (MOMENTUM-1) | 2030 | 2 (randomised) | [$^{177}$Lu]Lu-DOTATATE | 153 | Refractory grade 1-3 meningioma | None | Local SoC | RTOG Foundation, Inc. |
| NCT04903899 (LuDO-N) | 2031 | 2 | [$^{177}$Lu]Lu-DOTATATE | 24 | Paediatric recurrent or relapsed high-risk neuroblastoma | None | None | Jakob Stenman |
| NCT04082520 | 2031 | 2 | [$^{177}$Lu]Lu-DOTATATE | 41 | Grade 2-3 meningioma progressing after EBRT | None | None | Mayo Clinic |

EBRT: external beam radiation therapy; MTC: medullary thyroid cancer; NET: neuroendocrine tumour; SSTR: somatostatin receptor

**Supplementary Table S2.** Key phase 2 and 3 clinical trials investigating PSMA-targeted RLT in non-prostate malignancies registered on ClinicalTrials.gov.

| NCT Number | Estimated Completion, y | Phase | Radiopharmaceutical | Patients, n | Patient Population | Combination | Comparator Arm | Sponsor |
|---|---|---|---|---|---|---|---|---|
| NCT06959433 | 2028 | 3 | [$^{177}$Lu]Lu-PSMA-617 | 20 | Advanced or metastatic RCC | None | None | Ankara University |
| NCT04291300 (LUPSA) | 2023 | 2 | [$^{177}$Lu]Lu-PSMA-I&T | 12 | Recurrent or metastatic adenoid cystic carcinoma or salivary duct carcinoma | None | None | Radboud University Medical Center |
| NCT06852820 | 2027 | 2 | [$^{177}$Lu]Lu-PSMA-617 | 10 | Metastatic or unresectable HCC | None | None | Melissa Lumish |
| NCT06964958 (LASER) | 2028 | 2 | [$^{177}$Lu]Lu-PSMA-617 | 24 | Advanced or metastatic ccRCC | None | None | Dana-Farber Cancer Institute |
| NCT05867615. (LUBASKET) | 2028 | 2 | [$^{177}$Lu]Lu-PSMA-I&T | 83 | PSMA+ advanced or metastatic solid tumours progressing after standard treatment | None | None | Istituto Romagnolo per lo Studio dei Tumori Dino Amadori IRST S.r.l. IRCCS |
| NCT06783348 (RENALUT) | 2029 | 2 | [$^{177}$Lu]Lu-PSMA-617 | 48 | Advanced or metastatic ccRCC | None | None | European Organisation for Research and Treatment of Cancer - EORTC |
| NCT06322576 | 2035 | 2 | [$^{177}$Lu]Lu-PSMA-I&T | 10 | Recurrent or metastatic, inoperable adenoid cystic carcinoma | None | None | Sidney Kimmel Comprehensive Cancer Center at Johns Hopkins |

ccRCC: clear cell renal cell cancer; HCC: hepatocellular carcinoma; PSMA: prostate-specific membrane antigen; RCC: renal cell cancer

**Supplementary Table S3.** Clinical trials of FAP-targeted RLT agents registered on ClinicalTrials.gov.

| NCT Number | Estimated Completion, y | Phase | Radiopharmaceutical | Patients, n | Patient Population | Combination | Sponsor | Outcome Data |
|---|---|---|---|---|---|---|---|---|
| NCT05963386 | 2024 | 2 | [$^{177}$Lu]Lu-DOTA-EB-FAPI | 28 | Advanced or metastatic solid tumours | None | The First Affiliated Hospital of Xiamen University | ORR: 20% DCR: 65% |
| NCT04849247 | 2024 | 1 | [$^{177}$Lu]Lu-DOTA-FAPI | 30 | Advanced or metastatic solid tumours | None | The First Affiliated Hospital of Xiamen University | NA |
| NCT05432193 (FRONTIER)* | 2024 | 1 | [$^{177}$Lu]Lu-PNT6555 | 20 | Advanced or metastatic solid tumours | None | POINT Biopharma | NA |
| NCT05410821 | 2025 | 1 | [$^{177}$Lu]Lu-DOTA-EB-FAPI | 12 | RAIR thyroid cancer progressing after TKIs | None | The First Affiliated Hospital of Xiamen University | ORR: 25% DCR: 83% |
| NCT06553846 | 2025 | 1 | [$^{177}$Lu]Lu-FAP-75 | 50 | Advanced or metastatic solid tumours | None | Fudan University | NA |
| NCT05400967 | 2025 | 1 | [$^{177}$Lu]Lu-DOTA-EB-FAPI | 10 | Metastatic solid tumours | None | Peking Union Medical College Hospital | NA |
| NCT06081322 | 2025 | 1 | [$^{177}$Lu]Lu-DOTA-EB-FAPI | 29 | Metastatic PDAC or cholangiocarcinoma | Pembrolizumab | Zhejiang University | NA |
| NCT06636617 | 2025 | 1 | [$^{177}$Lu]Lu-JH04 | 9 | Metastatic solid tumours | None | First Affiliated Hospital of Fujian Medical University | NA |
| NCT05723640 | 2025 | 1 | [$^{177}$Lu]Lu-DOTA-EB-FAPI | 24 | Advanced or metastatic solid tumours | None | Yantai LNC Biotechnology Singapore PTE. LTD. | NA |

| | | | | | | | | |
|---|---|---|---|---|---|---|---|---|
| NCT06197139 | 2026 | 1 | [177Lu]Lu-XT117 | 20 | Advanced or metastatic solid tumours | None | Xinlu Wang | NA |
| NCT06211647 | 2026 | 1 | [177Lu]Lu-XT117 | 20 | Advanced or metastatic solid tumours | None | Ruimin Wang | NA |
| NCT06911489 | 2027 | 1 | [$^{177}$Lu]Lu-NRT6020 | 116 | Advanced or metastatic solid tumours | None | Chengdu New Radiomedicine Technology Co. LTD. | NA |
| NCT06898437 | 2027 | 1 | [$^{177}$Lu]Lu-DOTA-EB-FAPI | 20 | RAIR thyroid cancer progressing after TKIs | Everolimus | Peking Union Medical College Hospital | NA |
| NCT06880757 | 2028 | 2 | [$^{177}$Lu]Lu-FAP-2286 | 10 | Advanced or metastatic urothelial bladder cancer | Olaparib | Ankara University | NA |
| NCT04939610 (LuMIERE) | 2028 | 1/2 | [$^{177}$Lu]Lu-FAP-2286 | 222 | Phase 1: Advanced or metastatic solid tumours<br>Phase 2: PDAC, NSCLC, HR+/HER2- BC, HER2+ BC, TNBC | None | Novartis Pharmaceuticals | NA |
| NCT06640413 (Theratri) | 2028 | 1 | [$^{177}$Lu]Lu-OncoFAP-23 | 56 | Advanced or metastatic solid tumours | None | Philogen S.p.A. | NA |
| NCT07014254 | 2028 | 1 | [$^{177}$Lu]Lu-CTR-FAPI | 20 | Advanced or metastatic gastrointestinal and hepatobiliary malignancies | None | Xijing Hospital | NA |
| NCT06562192 | 2030 | 1 | [$^{177}$Lu]Lu-NNS309 | 124 | Advanced or metastatic PDAC, NSCLC, HR+/HER2-, TNBC, CRC | None | Novartis Pharmaceuticals | NA |

* terminated

BC: breast cancer; CRC: colorectal cancer; DCR: disease control rate; FAP: fibroblast activation protein; HER2: human epidermal growth factor receptor 2; HR: hormone receptor; NA: not available; NSCLC: non-small cell lung cancer; ORR: objective response rate; PDAC: pancreatic ductal adenocarcinoma; RAIR: radioiodine-refractory thyroid cancer; TKI: tyrosine kinase inhibitor; TNBC: triple negative breast cancer

**Supplementary Table S4.** Clinical trials of CAIX-targeted RLT agents registered on ClinicalTrials.gov.

| NCT Number | Estimated Completion, y | Phase | Radiopharmaceutical | Patients, n | Patient Population | Combination | Sponsor | Outcome Data |
|---|---|---|---|---|---|---|---|---|
| NCT00142415 | 2011 | 1/2 | [$^{177}$Lu]Lu-DOTA-girentuximab | 26 | Advanced or metastatic ccRCC | None | Ludwig Institute for Cancer Research | ORR: 4%<br>DCR: 78% |
| NCT00199875 | 2013 | 1 | [$^{90}$Y]Y-DOTA-girentuximab | 18 | Metastatic ccRCC | None | Ludwig Institute for Cancer Research | NA |
| NCT02002312 | 2015 | 2 | [$^{177}$Lu]Lu-DOTA-girentuximab | 14 | Advanced or metastatic ccRCC | None | Radboud University Medical Center | ORR: 7%<br>DCR: 64% |
| NCT05239533 (STARLITE 2) | 2026 | 2 | [$^{177}$Lu]Lu-DOTA-girentuximab | 41 | Advanced or metastatic RCC | Nivolumab | Memorial Sloan Kettering Cancer Center | NA |
| NCT05868174 (STARSTRUCK) | 2026 | 1 | [$^{177}$Lu]Lu-DOTA-girentuximab | 36 | Advanced or metastatic solid tumours | Peposertib | Telix Pharmaceuticals (Innovations) Pty Limited | NA |
| NCT05663710 (STARLITE 1) | 2027 | 1/2 | [$^{177}$Lu]Lu-DOTA-girentuximab | 100 | Advanced or metastatic ccRCC | Cabozantinib<br>Nivolumab | M.D. Anderson Cancer Center | NA |
| NCT06649682 (NYCRRLT) | 2027 | 1 | [$^{177}$Lu]Lu-NYM096 | 20 | Metastatic ccRCC | None | Peking Union Medical College Hospital | NA |
| NCT05706129 | 2029 | 1/2 | [$^{177}$Lu]Lu-DPI-4452 | 270 | Advanced or metastatic ccRCC, PDAC, CRC, UC | None | Debiopharm International SA | NA |

CAIX: carbonic anhydrase IX; ccRCC: clear cell renal cell cancer; CRC: colorectal cancer; DCR: disease control rate; NA: not available; ORR: objective response rate; PDAC: pancreatic ductal adenocarcinoma; UC: urothelial carcinoma

**Supplementary Table S5.** Clinical trials of GRPR-targeted RLT agents registered on ClinicalTrials.gov.

| NCT Number | Estimated Completion, y | Phase | Radiopharmaceutical | Patients, n | Patient Population | Combination | Sponsor |
|---|---|---|---|---|---|---|---|
| NCT03872778 (NeoRay) | 2026 | 1/2 | $[^{177}Lu]$Lu-NeoB | 51 | Advanced or metastatic solid tumours | None | Advanced Accelerator Applications |
| NCT05633160 (COMBAT)* | 2026 | 1/2 | $[^{67}Cu]$Cu-SAR-BBN | 38 | Progressive mCRPC not eligible for PSMA RLT | None | Clarity Pharmaceuticals Ltd |
| NCT05283330 | 2027 | 1 | $[^{212}Pb]$Pb-DOTAM-GRPR1 | 55 | Advanced or metastatic GRPR+ solid tumours | None | Orano Med LLC |
| NCT06379217† (NEPC) | 2027 | 1 | $[^{177}Lu]$Lu-NeoB | 36 | Metastatic NEPC | None | Novartis Pharmaceuticals |
| NCT05739942 | 2031 | 1/2 | $[^{177}Lu]$Lu-NeoB | 48 | Group 1: newly diagnosed GBM<br>Group 2: recurrent GBM | Group 1: RT and temozolomide<br>Group 2: none | Novartis Pharmaceuticals |
| NCT06247995 (NeoB-Cap1) | 2031 | 1/2 | $[^{177}Lu]$Lu-NeoB | 58 | ER+, HER2- metastatic BC | Capecitabine | Novartis Pharmaceuticals |
| NCT05870579 | 2032 | 1 | $[^{177}Lu]$Lu-NeoB | 48 | Advanced or metastatic ER+ HER2- BC | Ribociclib<br>Fulvestrant | Novartis Pharmaceuticals |

* terminated

† adaptive trial in NEPC assessing PSMA, SSTR2, and GRPR expression with PET imaging; patients receive RLT directed to the predominant target, which may include GRPR

BC: breast cancer; ER: estrogen receptor; GBM: glioblastoma; GRPR: gastrin-releasing peptide receptor; HER2: human epidermal growth factor receptor 2; mCRPC: metastatic castration resistant prostate cancer; NEPC: neuroendocrine prostate cancer; PSMA: prostate-specific membrane antigen; RLT: radioligand therapy; RT: radiotherapy
